# High prevalence of elevated blood lead levels in both rural and urban Iowa newborns: Spatial patterns and area-level covariates

**DOI:** 10.1371/journal.pone.0177930

**Published:** 2017-05-16

**Authors:** Margaret Carrel, David Zahrieh, Sean G. Young, Jacob Oleson, Kelli K. Ryckman, Brian Wels, Donald L. Simmons, Audrey Saftlas

**Affiliations:** 1 Department of Geographical & Sustainability Sciences, University of Iowa, Iowa City, IA, United States of America; 2 Department of Epidemiology, University of Iowa, Iowa City, IA, United States of America; 3 Department of Biostatistics, University of Iowa, Iowa City, IA, United States of America; 4 Department of Pediatrics, University of Iowa, Iowa City, IA, United States of America; 5 State Hygienic Laboratory, University of Iowa, Iowa City, IA, United States of America; Stony Brook University, Graduate Program in Public Health, UNITED STATES

## Abstract

Lead in maternal blood can cross the placenta and result in elevated blood lead levels in newborns, potentially producing negative effects on neurocognitive function, particularly if combined with childhood lead exposure. Little research exists, however, into the burden of elevated blood lead levels in newborns, or the places and populations in which elevated lead levels are observed in newborns, particularly in rural settings. Using ~2300 dried bloods spots collected within 1–3 days of birth among Iowa newborns, linked with the area of mother’s residence at the time of birth, we examine the spatial patterns of elevated (>5 μg/dL) blood lead levels and the ecological-level predictors of elevated blood lead levels. We find that one in five newborns exceed the 5 μg/dL action level set by the US Centers for Disease Control & Prevention (CDC). Bayesian spatial zero inflated regression indicates that elevated blood lead in newborns is associated with areas of increased pre-1940s housing and childbearing-age women with low educational status in both rural and urban settings. No differences in blood lead levels or the proportion of children exceeding 5 μg/dL are observed between urban and rural maternal residence, though a spatial cluster of elevated blood lead is observed in rural counties. These characteristics can guide the recommendation for testing of infants at well-baby appointments in places where risk factors are present, potentially leading to earlier initiation of case management. The findings also suggest that rural populations are at as great of risk of elevated blood lead levels as are urban populations. Analysis of newborn dried blood spots is an important tool for lead poisoning surveillance in newborns and can direct public health efforts towards specific places and populations where lead testing and case management will have the greatest impact.

## Introduction

Lead is a ubiquitous and highly potent human toxicant that readily crosses the placenta of the developing fetus, and impairs the development and function of multiple organ systems [1, 2]. The physiologic stress of pregnancy can stimulate mobilization of lead from bone into maternal blood, particularly when calcium demands are high [3]. Hence, maternal bone stores of lead represent an endogenous reservoir and potential source of fetal lead exposure. Factors that may increase transfer of lead to the fetus include high maternal blood pressure [2, 4], low calcium levels and milk intake [5], low hemoglobin levels and anemia [3], and alcohol intake in the third trimester of pregnancy [6]. Environmental and lifestyle exposures (e.g. leaded paint or pipes in older houses, leaded gasoline, leaded soil or dust or air pollution, leaded food and cosmetic products, cigarette smoke, consumption of fish) also influence maternal, and, by extension, fetal blood lead levels [7–10].

It is well established that childhood lead exposure causes impairments in central neurological function, increasing the risk of a wide spectrum of developmental delays, intellectual deficits, and behavioral problems [11–14]. These developmental delays associated with higher lead levels are observable within hours following birth [15]. Greater neurocognitive effects are seen in children with blood lead levels of 5–10 μg/dL or more, as compared to those with levels of 1–2 μg/dL [16–18]. However, *in utero* exposure to maternal blood lead levels <10 μg/dL produces neurotoxic effects at the lowest levels studied [7, 19–21]. For this reason, in 2012 the Centers for Disease Control & Prevention (CDC) lowered the level of concern for blood lead from >10 μg/dL to >5 μg/dL.

Despite dramatic declines in blood lead levels in all segments of the population over the past several decades, disparities in the burden of lead exposure persist with levels highest among low-income families, those living in pre-1940s housing, non-Hispanic blacks, and immigrants from countries where leaded gasoline and lead-containing consumer products are in current or recent use [22–25]. Exposure to lead can also come via contaminated outdoor soils, from legacy sources of lead such as leaded gasoline, or via re-suspended particulate matter in air pollution [26–28]. A strong seasonal component to atmospheric and soil exposure has been shown, leading to elevated blood lead levels in children exposed to greater amounts of leaded soil and dust and particulate matter during warmer months [29–33]. Recent events, namely in Flint, Michigan, have brought lead poisoning back into the consciousness of the general public [34]. Though newborn infants are at risk for the adverse effects of lead exposure and poisoning the primary focus in the public health and research communities is on exposure and poisoning in older children.

The literature examining lead exposure or poisoning in childhood focuses predominantly on urban communities [26, 32, 35–49]. Few studies examine rural inhabitants, particularly rural children, and those that do often find lower lead exposure or lower blood lead levels in rural versus urban populations [24, 50–58]. While the density of children exposed to lead in rural areas is lower than that in urban areas, the potential for exposure is still high as many of the same environmental and lifestyle risk factors are at play in rural areas. Approximately one third of Iowa’s residents live in rural areas, though these rural areas comprise the majority of the landmass of Iowa.

This study, utilizing data from newborns in Iowa, seeks to understand the rural/urban variation in lead levels observed in newborns, as well as the spatial patterns of elevated blood lead levels and their area-level covariates. In doing so, we identify the prevalence of Iowa newborns with lead levels that require case management, detect any differences in rural versus urban patterns of elevated blood lead and explore places and populations where elevated blood lead levels are most likely to occur.

## Materials and methods

To determine blood lead levels in Iowa newborns, newborn dried blood spot samples were collected as part of routine newborn screening over a 5-month period in 2006 and sequentially analyzed at the State of Iowa Hygienic Laboratory. Two, 1/8-inch diameter punches were obtained using a Wallac Multipuncher (Model# 1296–081). Samples were transferred to 96-well microfilter plates (Millipore, Cat. #MSBVN1210) and extracted using a solution containing 1% tetramethylammonium hydroxide, 1% isopropyl alcohol, and 0.1% ammonium pyrrolidine dithiocarbamate. Extraction was accomplished using mild agitation on a table shaker for one hour followed by filtration. Small well volumes afforded by the microfilter plates limited the sample volume to 0.3 mL allowing for only one analysis per extraction. The blood volume was estimated to be 6 μL resulting in a 1:50 dilution of the blood. Lead analysis was conducted using inductively coupled plasma mass spectrometry (ICP-MS, PerkinElmer Elan DRC II). The signals from multiple lead isotopes (206, 207, and 208) were summed to account for natural variations in isotopic abundances. A CLIA reviewed routine whole blood analysis methodology was employed to analyze the blood samples by ICP-MS. The instrument was calibrated using external standards prepared with whole blood and extraction solution in approximately the same ratio as specimens. All measurements are reported in units of micrograms (μg) per deciliter (dL).

This project was deemed not human subjects research by the University of Iowa IRB. No identifiers, such as complete residential address at time of birth, were associated with the dried blood spots and they were collected as part of a routine state newborn metabolic disorder surveillance program.

Exclusions from the analysis were made for the following reasons: blood samples from three plates contained negative values, thus all samples from those plates were excluded from the analysis (n = 92); any samples with relative standard deviations of >10%, an indicator of poor precision or of levels at or below the detection limit, were also discarded (n = 18); one sample had a blood lead level of >80 μg/dL and was deemed an extreme outlier. Blood samples missing the mother’s ZIP code of residence were excluded as many women in Iowa travel outside of their ZIP code area to give birth, particularly rural women—thus the ZIP code of the hospital where the delivery took place would not accurately represent the population or environment where the mother resides (n = 181).

Blood lead levels in newborns were assigned to the ZIP code of mother’s residence, and then further classified into ZIP code tabulation areas (ZCTAs). ZCTAs represent aggregations of Census block groups where the majority of residents share the same ZIP code, and are generated by the Census Bureau to allow comparison of ZIP code data to sociodemographic data collected in Census-defined geographies. ZCTAs were further classified as urban or rural to explore potentially different spatial patterns and ecological covariates. A ZCTA was designated as urban if it intersected with the urbanized areas of Iowa, as defined by the 2010 Census (US Census Bureau) or as rural if they did not intersect with urbanized areas. Urbanized areas are defined by the Census as having a population of >50,000 with a density of at least 500 people per square mile. The assignment of urban/rural status was completed in ArcMap 10.3 (Esri, Redlands, CA).

The outcome variable of interest is the count of blood samples in a ZCTA that are >5 μg/dL, the reference level currently recommended by the US CDC. Additionally, a level of >10 μg/dL was used based upon the reference level recommended by the CDC at the time the blood samples were taken[59].

The Wilcoxon-Mann-Whitney test was used to detect significant differences in the distribution of blood lead levels between rural and urban newborns and a chi-square test was used to detect differences in the number of rural versus urban children whose blood lead levels exceeded the 5 μg/dL or >10 μg/dL reference levels set by the CDC. Statistical calculations were carried out in R. P-values are two-sided. P-values less than 0.05 were deemed statistically significant.

Descriptive mapping of the locations of births as well as the associated lead levels were created in ArcMap 10.3. Points were randomly generated within each ZCTA equal to the number of samples in that ZCTA, and were distributed within ZCTA bounds so that areal summary measures would be avoided in mapping and spatial analysis. These random points were further used for hotspot analysis to explore whether observations of elevated lead levels were randomly spatially patterned or clustered in certain neighboring ZCTAs. The continuous value of blood lead was used as the outcome variable rather than a dichotomous measure of above/below the CDC threshold. Optimized hotspot analysis (OHSA) is a tool designed to identify significant spatial clusters of high or low values. OHSA corrects for spatial dependence within data and for multiple testing. Given that the presence of spatial clusters could be influenced by the assignment of lead levels to the randomly distributed points within ZCTAs, we randomly permuted the assignment of points in each ZCTA and iterated the OHSA one thousand times. Hotspots that persisted in space even when points were permuted (present >950 times) were deemed to be authentic hotspots rather than an artifact of the arbitrary spatial distribution of data points within ZCTAs.

To explore how variation in ZCTA-level ecological variables is associated with the spatial variation in counts of elevated lead levels (>5 μg/dL or >10 μg/dL) in newborn blood samples, we undertook a Bayesian disease mapping analysis. See [60] for specifics on Bayesian disease mapping spatial models. Nearly half (449) of the 935 ZCTAs in Iowa did not experience a newborn during the study design’s 5-month data collection window. If no newborns occurred within a ZCTA, then the ZCTA was not eligible for a non-zero response (i.e. count >5 μg/dL). These responses are referred to as structural zeros. Of the remaining 486 ZCTAs that experienced a newborn with assessable blood samples during the data collection window, many had zero or one sample exhibiting elevated lead levels, indicating that the counts of elevated lead levels is governed by a Poisson (λ) distribution. Therefore, our data arise from two zero generating processes. In one process, the outcome is always a zero count, while in the other process the counts, some of which may be zero, follow a standard Poisson process.

To account for these two generating processes, a spatial zero-inflated Poisson (ZIP) regression model, which allows for an over-abundance of zero counts and includes spatial random effects, was used to fit the data using Bayesian methods [61]. In other words, with probability p we sample a degenerate distribution at 0 and with probability (1 –p) we sample a Poisson (λ) distribution. In our spatial ZIP model, log (λ) is assumed to be a linear function of a set of regional level covariates and a spatial random effect while log (p / (1-p)) is assumed; initially log (p / (1-p)) was assumed to be a linear function of the regional covariates, however, the estimated posterior distributions of the coefficients associated with those covariates covered a wide range and included zero. Since we know the proportion of ZCTAs (48.0%) that did not experience a newborn during the study design’s 5-month data collection, this information was included in the prior distribution placed on each *p*_*i*_ (*i* = 1,…,935) in the spatial ZIP model. As a sensitivity analysis, the Bayesian analysis was repeated assuming standard Poisson regression with inclusion of spatial random effects. For that, the 449 ZCTAs were treated as missing counts rather than structural zeros and the missing counts were incorporated in the joint posterior distribution.

The objective of the Bayesian spatial ZIP regression analysis is to determine which of several regional level covariates, when assessed in the presence of each other, predict the presence of elevated blood lead levels (>5 μg/dL or >10 μg/dL) in Iowa newborns. The right hand side of the model is a function of 6 regional level covariate effects, an offset, and a random spatial effect. Data on hypothesized covariates of newborn blood lead levels were acquired from the 2007–2011 American Community Survey (ACS). While the newborn lead data was collected in 2006, ACS data is not available at the ZCTA scale for that year. The ACS from 2007–2011 was chosen over the decadal census from 2000 or 2010 as better representing the population being studied in 2006. The potential for household exposure to lead via lead-based paint, was assessed via the inclusion of the percentage of houses in the ZCTA built prior to 1940. While lead was phased out of paint products in the 1950s-1970s and banned in the US in 1978, research in the US indicates that houses built prior to 1940 are associated with much higher risk of lead exposure than those built in following decades [62–64]. The potential for maternal exposure in countries where environmental lead exposure is more prevalent is captured by the percent of women in a ZCTA who were foreign born. Low education and income are often predictors of high lead exposure. Thus, the percentage of women of reproductive age (i.e., ages 18–44) living in poverty and the percentage who had less than a college education are calculated for each ZCTA from the Census ACS data. Median household income in each ZCTA is also taken from the ACS. Urban and rural designation for each ZCTA, was also included as it was hypothesized to delineate risk of lead poisoning. To control for greater numbers of samples potentially occurring in ZCTAs where there were greater numbers of women of reproductive age, the total number of women ages 18–44 was calculated for each ZCTA and the natural logarithm of the corresponding total was included in the model as an offset variable. For the outcome variable (>5 μg/dL or >10 μg/dL), further exploratory analyses included the assessment of all two-way interactions between the designation of urban and rural and one of the other 5 explanatory variables. The goal was to ascertain if the magnitude of the effect for each explanatory variable was different according to the rural and urban designation of the ZCTA.

The distribution of three of the covariates did not deviate from a normal distribution. These three covariates were centered and standardized prior to inclusion in the spatial ZIP model. The skewed right distribution of the percent women in poverty was dichotomized at the median (1 if > 11.51%; 0 otherwise). As the covariate for being foreign born was severely skewed to the right, it was dichotomized at the 75th percentile (1 if > 2.45%; 0 otherwise). Seven ZCTAs were missing regional covariate information from the ACS. Single imputation was used on a variable by variable basis, assuming missing at random. The imputation models used were predictive mean matching and logistic regression for the continuous covariates and dichotomous covariates, respectively.

To analyze spatial effects, we used a conditional autoregressive (CAR) model. Specifically, we used the intrinsic CAR model to specify the spatial association, which includes a precision parameter. Spatial association is defined through a neighborhood structure where one region is related with other regions that share a common border, determined by an adjacency matrix (first order Queen’s contiguity) generated in ArcMap 10.3.

Priors were chosen to ensure a proper posterior distribution. Relative vague Normal priors were chosen for the covariate parameters, and the spatial standard deviation prior was uniform (0,100). A Beta(2, 2) prior (i.e. with mean 0.5) was placed on each of the *p*_*i*_ (*i* = 1,…,935). Posterior summary statistics are based on three chains with 50,000 iterations per chain and a 25,000 burn-in period for each chain. To decrease autocorrelation, samples were thinned, using only every tenth step in the sampler. The model was programmed using OpenBUGS [65, 66].

## Results

**Fig 1:**
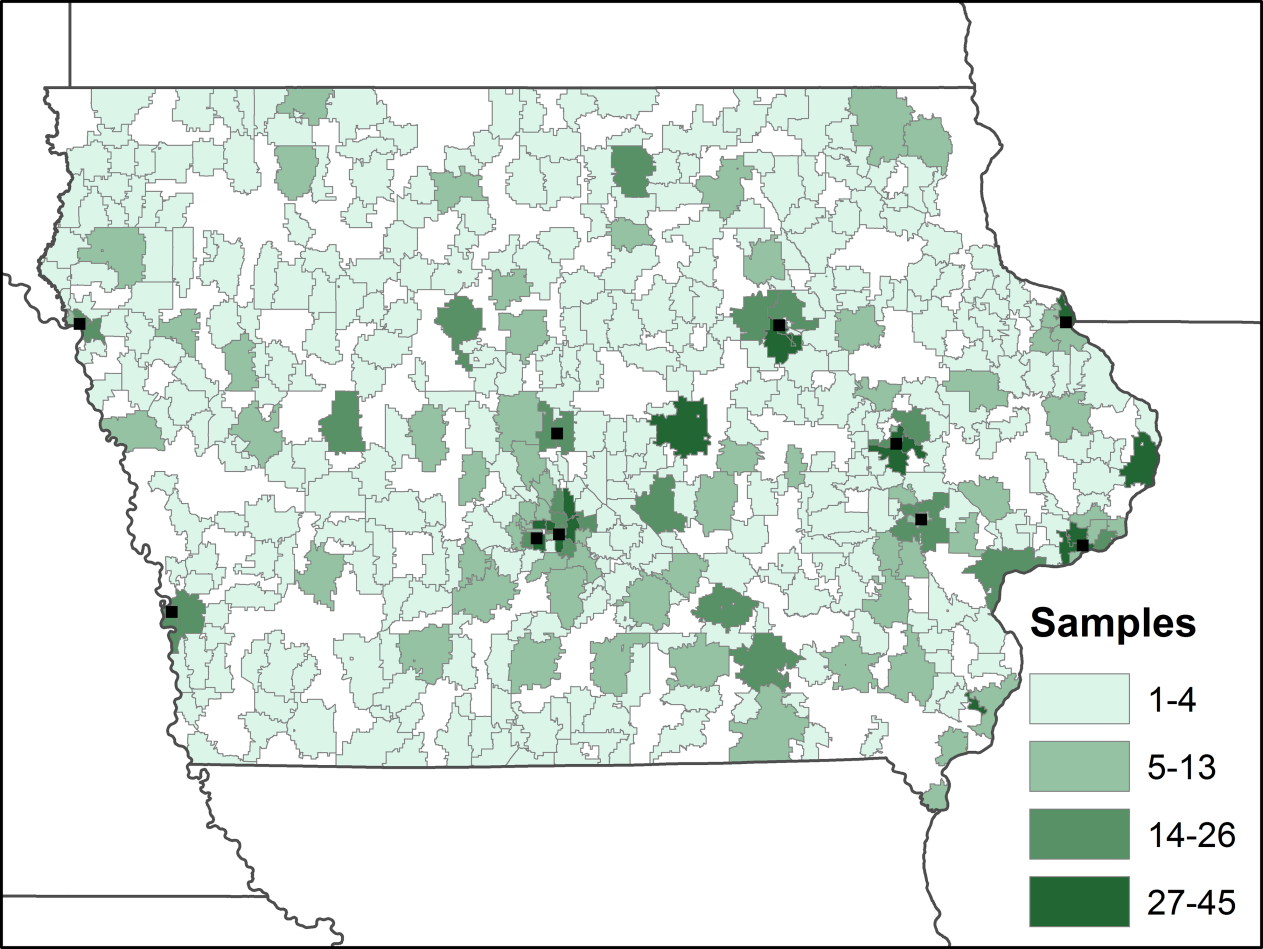
Number of samples per ZCTA included in the study (areas of white represent ZCTAs with no samples). The location of urban areas in Iowa with populations over 50,000 people are shown for reference.

There are a total of 2376 observations in our final sample (S1 File). For each case, we identified the ZCTA of residence among the 935 ZCTAs in Iowa. There were true-zero observations in 449 (48.0%) of the ZCTAs, with a mean of 4.9 and median of 2.0 observations per ZCTA (Fig 1).

**Fig 2:**
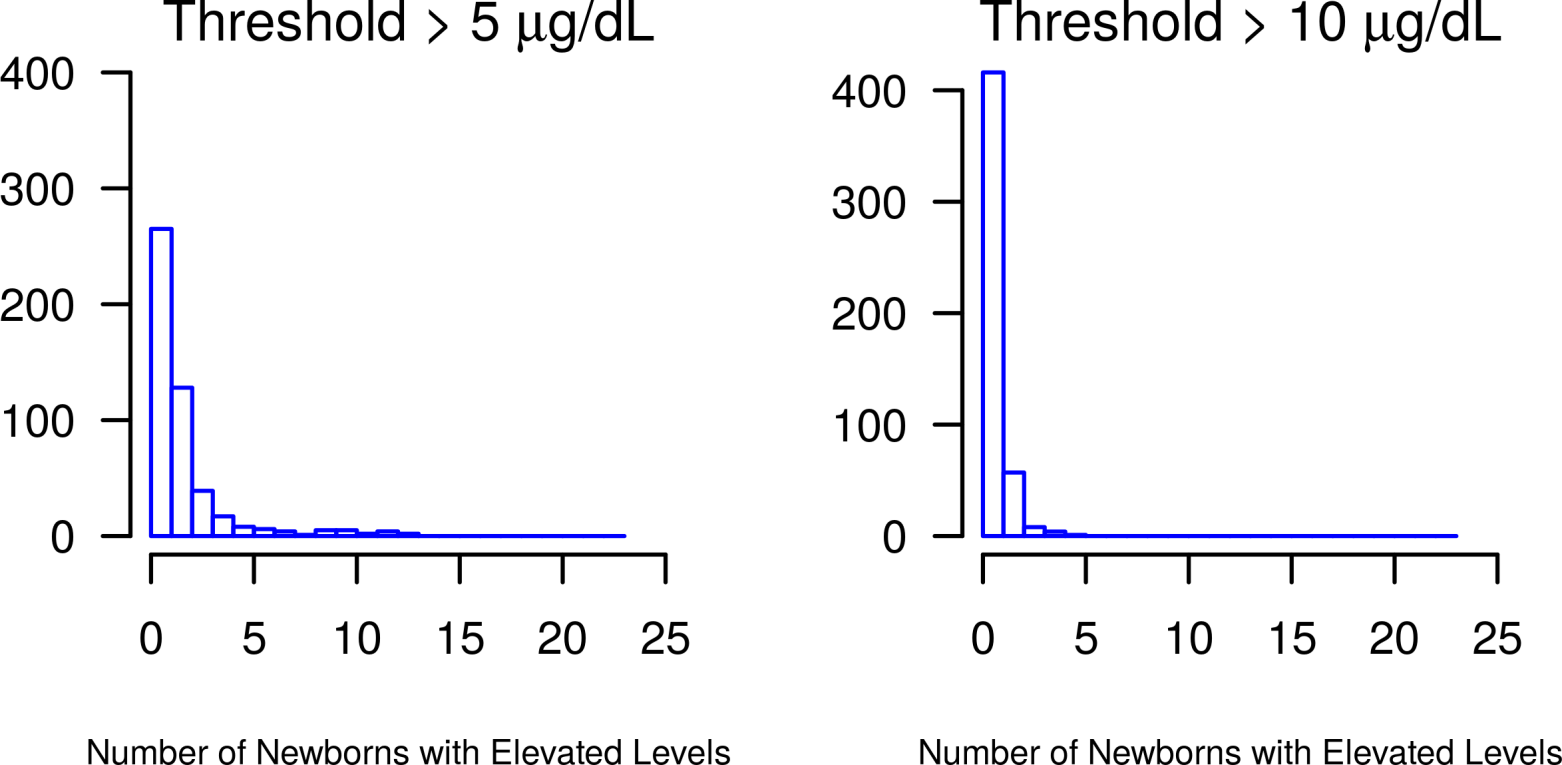
Distribution of ZCTAs with samples exhibiting elevated lead levels. The majority of ZCTAs had zero samples above the 5 μg/dL and 10 μg/dL thresholds.

The median [minimum, maximum] lead level was 2.47 μg/dL [0.09, 39.19] (Fig 2). The lower and upper quartiles were 1.53 μg/dL and 4.59 μg/dL, respectively, while the mean (standard deviation) lead level was 3.57 μg/dL (3.382). There were 22.0% (n = 523) of the samples with lead levels above the clinically relevant reference level >5 μg/dL and 3.7% (n = 89) of samples above the reference level >10 μg/dL.

**Fig 3:**
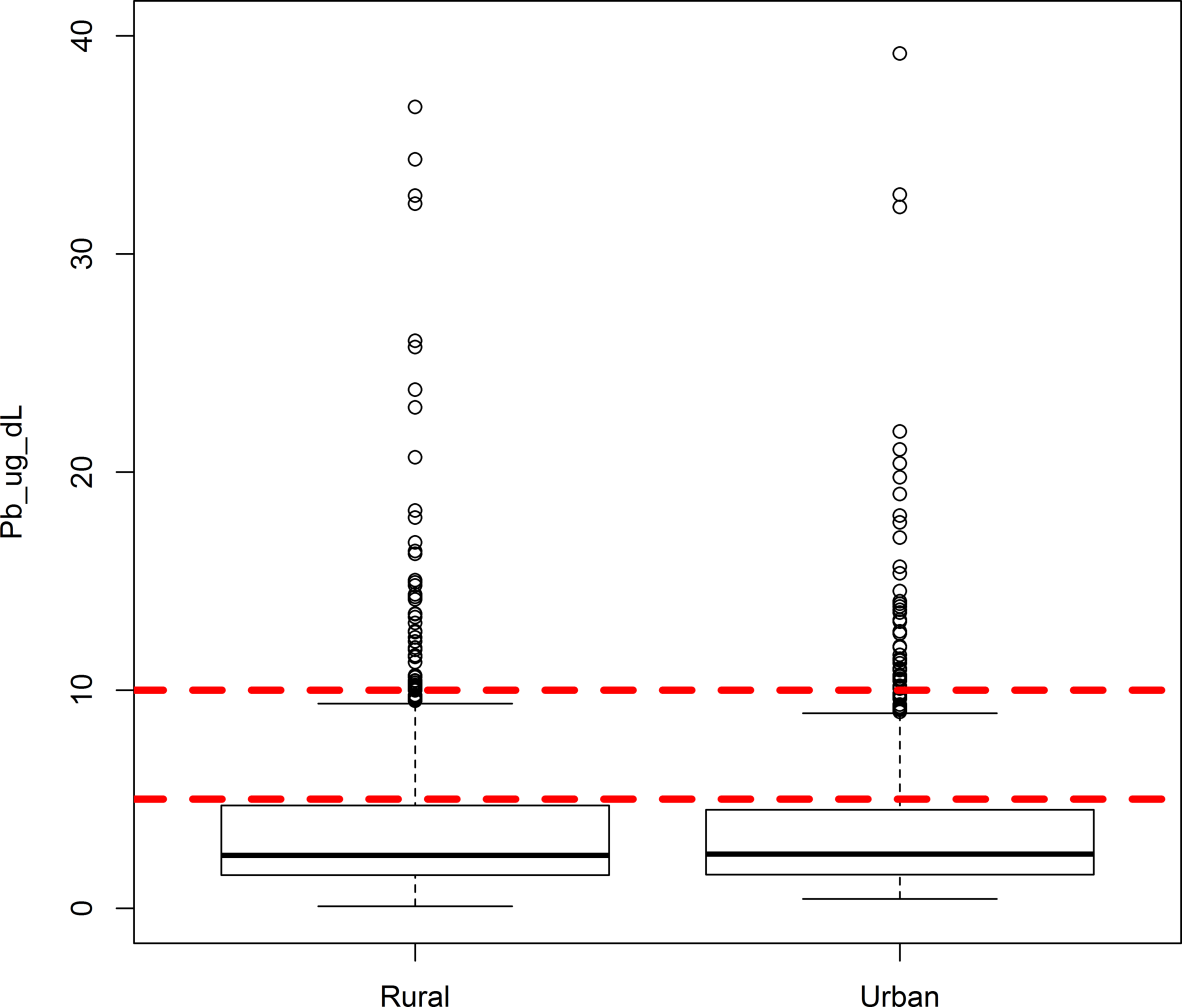
Distribution of blood lead levels in Iowa newborns stratified by mother’s residence in rural and urban ZCTAs. Current and previous CDC reference levels of 5 μg/dL and 10 μg/dL are included for context.

Plotting the distribution of blood lead levels in newborns according the urban/rural status of the ZCTA of mother’s residence indicated a high degree of similarity (Fig 3). A Wilcoxon-Mann-Whitney test found no significant difference in continuous lead values between urban and rural areas (p = 0.97). Similarly, a chi-square test found no significant differences in the number of urban (261/1195) versus rural children (262/1181) whose blood lead levels exceed 5 μg/dL (p = 0.87). No significant difference was found for urban (43/1195) versus rural (45/1181) children with lead levels exceeding 10 μg/dL (p = 0.78).

**Fig 4:**
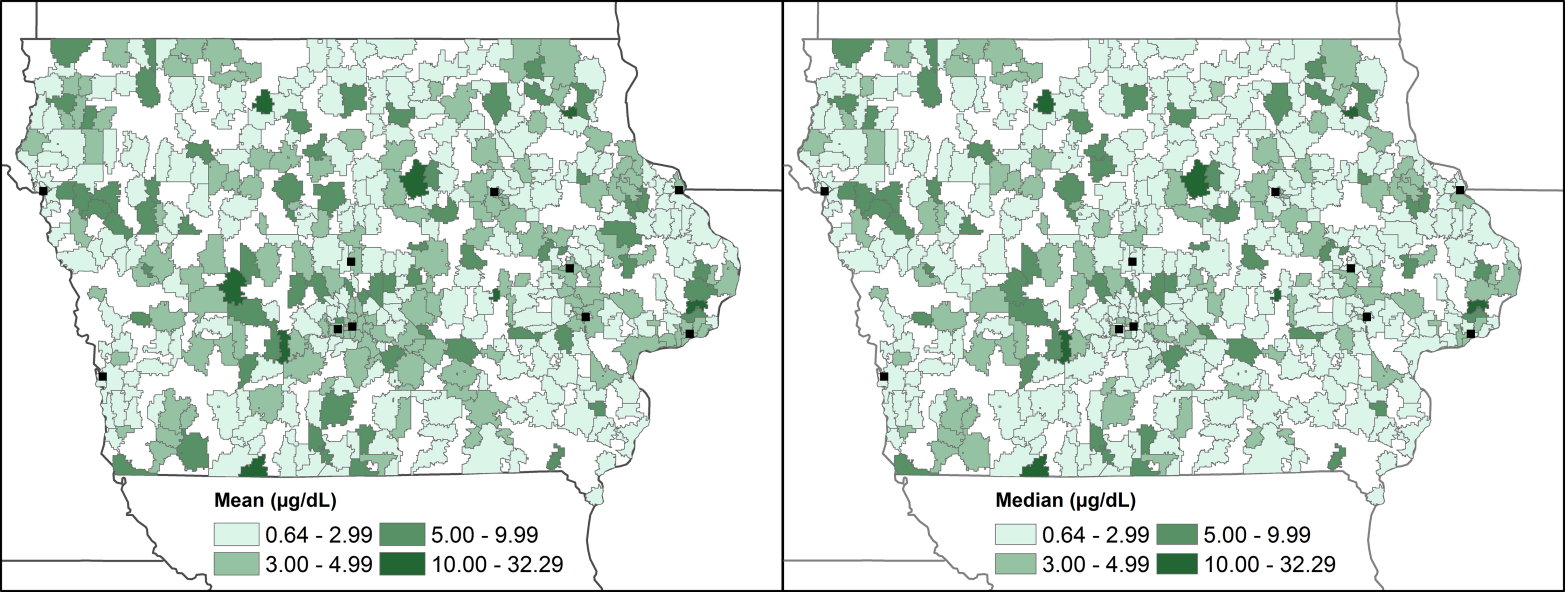
Spatial patterns of mean and median blood lead levels in Iowa newborns included in the study. The location of urban areas in Iowa with populations over 50,000 people are shown for reference.

**Fig 5:**
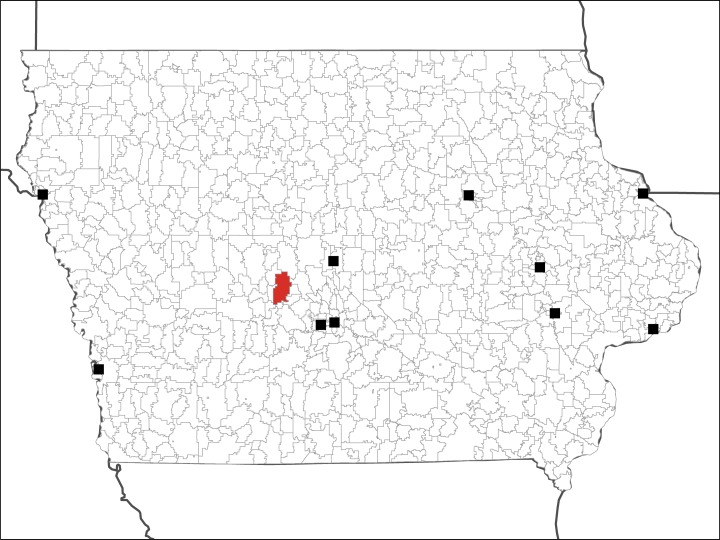
ZCTA with blood lead observations belonging to hotspots, persistent over random permutations of points. The location of urban areas in Iowa with populations over 50,000 people are shown for reference.

The spatial distribution of ZCTA-level mean and median blood lead levels do not suggest strong spatial patterning (Fig 4). ZCTAs with low mean and median levels are neighbored by those with high lead levels. When random points representing individual newborns are permuted inside of ZCTAs, an area in the central western portion of the state has a hotspot of high blood lead levels (Fig 5). This hotspot is persistent across the random redistribution of points within ZCTAs, and was detected 972/1000 times (quasi p-value = 0.028). Located in a rural ZCTA, the observations (n = 10) within this ZCTA had lead levels ranging from 1.5 μg/dL to 36.7 μg/dL, and a mean of 9.94 μg/dL and median of 4.74 μg/dL.

Assuming a spatial ZIP regression model, univariate analysis of the hypothesized regional covariates with the outcome of >5 μg/dL of lead in newborn blood samples were similar to the multivariate analysis (data not shown). When all 6 regional covariates are included in the Bayesian spatial ZIP regression model for the outcome of >5 μg/dL the variables maternal poverty, foreign born, and median income are not significant predictors of lead levels based on the 95% credible interval (i.e. the 95% credible intervals include zero) (Table 1). Adjusting for the other covariates, ZCTAs with a higher proportion of women with less than a college degree are associated with elevated blood lead levels. Similarly, after controlling for the other covariates, ZCTAs with an increased proportion of pre-1940s housing are more likely to have elevated blood lead levels. While inference was not sensitive to other functional forms of the covariate percent in poverty, inference was sensitive to dichotomizing the covariate percent foreign-born at two other cut-points explored, namely, at the 25th and 50th percentiles (0% and 0.7%, respectively). The estimated coefficient remained negative, but the 95% credible interval excluded zero.

**Table 1:** Spatial zero-inflated Poisson regression of hypothesized area covariates for the outcome of counts of blood lead levels >5 μg/dL in ZCTAs.

Variable Description	Counts of Lead Levels > 5 μg/dL	
	Mean (SD)^e^	95% Credible Interval^e^
Intercept	-6.3160 (0.1249)	[-6.5620, -6.077]
% of Childbearing Women in Poverty^a^	-0.1153 (0.1416)	[-0.3921, 0.1628]
% of Childbearing Women Foreign Born^b^	-0.1563 (0.1388)	[-0.4269, 0.1159]
% of Housing Built Before 1940^c^	0.1609 (0.0733)	[0.0157, 0.3035]
% of Childbearing Women with Less Than a College Education^c^	0.2391 (0.0861)	[0.0713, 0.4084]
Median Household Income^c^^,^^d^	0.0242 (0.0734)	[-0.1187, 0.1704]
Urban vs. Rural	-0.1196 (0.1785)	[-0.4785, 0.2237]
Spatial Standard Deviation	0.5699 (0.1785)	[0.2701, 0.8580]

^a^ The variable percent of childbearing women in poverty was treated as a categorical variable in the analysis; (1 if the percent was greater than the 50th percentile of 11.51% and 0 otherwise).

^b^ The variable percent of childbearing women who were foreign born was treated as a categorical variable in the analysis; (1 if the percent was greater than the 75th percentile of 2.45% and 0 otherwise).

^c^ The other 3 covariates were treated as continuous covariates, centered and standardized to obtain model stability.

^d^ The covariate median household income is per $10000.

^e^ % = Percent. SD = Standard Deviation. Posterior summary statistics are based on three converged chains with 10,000 iterations per chain, with the first 5,000 iterations discarded as a burn-in period. To decrease autocorrelation, samples were thinned, using only every fiftieth step in the sampler.

While assuming a spatial ZIP model, we performed separate exploratory analyses that examined how the urban/rural variable interacted with each of the other 5 explanatory variables. Each model comprised the explanatory variable urban/rural, one other explanatory variable, and the two-way interaction. The estimated posterior distribution for the coefficient associated with each interaction term was variable and crossed zero. Thus, rural or urban designation was not a significant predictor of increased counts of lead levels >5 μg/dL in ZCTAs.

For the outcome of >10 μg/dL counts in ZCTAs, none of the coefficients were well estimated; all estimated posterior distributions covered a wide range and crossed zero.

## Discussion

A large portion of the newborns sampled in this study had blood lead levels that were higher than the CDC guidelines set in 2012. There is no safe level of lead as even very small amounts are able to cause neurocognitive defects, but amounts >5 μg/dL are now recommended to receive case management by health professionals [67]. Approximately one in five newborns included in the study, with similar distribution in rural and urban areas of the state, had blood lead concentrations above the action level.

The >5 μg/dL action level was determined using the top 2.5% of lead levels observed in children in the 2007–2008 and 2009–2010 National Health and Nutrition Examination Survey (NHANES). Other studies, however, have observed blood lead levels >5 μg/dL in over ten percent of children tested (without lead pipes servicing the home) and in over twenty-five percent of children tested (with lead servicing the home) [36]. Thus, while our study identifies a much higher prevalence of elevated blood lead levels in Iowa newborns it is not outside the realm of possibility. It should also be noted that the CDC action level is for children ages 1–5 and our study population is composed of newborn infants less than 3 days old. Blood lead levels have been shown to vary, both increasing and decreasing, with repeat measurements from birth through older ages [68]. Additionally, the measures of blood lead concentrations included in this study are from dried blood spots rather than whole blood measures. Research indicates, however, that dried blood spot levels are correlated with whole cord blood levels under careful sampling procedures (data in preparation) and with venous whole blood samples in both hospital and community collection settings [69, 70].

The potential for lead contamination of the dried blood spot cards, as has been observed in other studies, cannot be ruled out though precautions were taken to prevent contamination [71–75]. Microfilter plates were rinsed with 2% nitric acid prior to use and reagents were screened for lead contamination. Dried blood spots were handled with Teflon®-coated forceps. Blank filter punches were randomly selected for analysis to determine the potential presence of lead in filter paper. Of 184 blank filters analyzed, the majority (167/184, 90.8%) were <1.0 μg/dL and all but two were less than 4 μg/dL and (Table 2). The two which exceed 4 μg/dL were determined to be 5.3 and 15.8 μg/dL. However, contamination and other random errors are expected to exhibit non-differential spatial bias, in that the samples are tested as they are delivered to the hygienic laboratory and are not run only with other samples from the hospital of delivery. Thus, any contamination present in the study is not disproportionately associated with a specific region of the state, or the sociodemographic covariates from that region. Additionally, blood spots are an established way to screen for elevated blood lead levels, and the filter paper used in the study meets the approval standards for statewide newborn screening [76, 77].

**Table 2:** Distribution of lead values in blank filter paper used for analysis.

Number of filter blanks	Pb range (μg/dL)
80	< 0.1
39	0.1 to < 0.2
48	0.2 to < 1.0
6	1.0 to < 2.0
4	2.0 to < 3.0
5	3.0 to < 4.0
2	> 4.0

The NHANES sample population is drawn primarily from urban populations, and our analysis is comprised of approximately equal numbers of rural and urban newborns [78]. Thus, we may be capturing a larger number of newborns with risk exposure in rural environments than nationally representative NHANES data. Our analyses identified a single hotspot in Iowa where the lead levels for newborns in ZCTAs are consistently elevated; this hotspot is found in a rural ZCTA in the west-central portion of the state, crossing the boundary between Boone, Dallas and Green counties. Data on elevated lead levels in children in Iowa counties reported to the CDC in 2006, the same year as the current study’s cohort was obtained, indicates that these three counties had case rates of 0.8%-1.6%, higher than many other Iowa counties, but were not among the counties with the top five case rates [79].

Most studies that have examined urban versus rural differentials in blood lead levels in children have found urban residence to be associated with higher lead levels or greater numbers of children exceeding a risky threshold level. One study, in North Carolina, found rural residence to be a significant risk factor for having blood lead levels of >15 μg/dL and that this effect was stronger for black males [55]. Prior research in Canada has indicated similar frequency distributions of cord blood lead levels in newborns across the rural/urban divide but higher numbers of urban children with elevated cord blood lead [80]. We find that not only is the frequency distribution of blood lead levels between rural and urban Iowa newborns similar but so too are the numbers of children exceeding the CDC action level of 5 μg/dL. Due to data limitations we are unable to explore, as the North Carolina researchers did, differentials in the impact of rural residence by individual-level sociodemographic characteristics. It should be noted that the designation of rural versus urban in this research differs from that of the North Carolina research in that the classification used in the current study is at the ZCTA rather than the county scale.

Characteristics of the population of ZCTAs where there were higher counts of newborns with lead levels above the 5 μg/dL threshold included higher percentages of women with less than a college education and with a higher proportion of older housing. Older housing stock in both rural and urban Iowa is potentially associated with exposure to not only lead paint but also lead piping, a known risk factor for elevated blood lead levels [36]. The Midwest has some of the oldest housing stock in the US, and rural areas in particular have typically older housing [81]. Analysis of NHANES (1999–2004) data indicated that Midwestern children had the highest percentage of elevated blood lead levels [82] NHANES data also indicate that blood lead levels are inversely related to educational attainment[83]. Women with lower educational status may be less aware of the dangers of trans-placental exposure to lead contamination during pregnancy, engage in other risky behaviors such as smoking, or have less agency to decrease exposure to sources of environmental contamination (i.e. less ability to paint over leaded paint, to test for lead in water and replace pipes, move to another residential location away from legacy lead in soil, etc.). Estimates of lead paint in households indicate that renter occupied, low income and minority households, characteristics that are often spatially collinear with educational attainment, have higher percentages of lead contaminated units [62].

The median household income, percentage of women living in poverty, and percentage of foreign-born women were not significantly associated with elevated lead in newborn blood samples. There was no difference in significant predictors according to rural or urban maternal residence, suggesting that the same risk factors apply regardless of population density. No covariates were significantly associated with increased counts of blood lead levels >10 μg/dL. This is likely due to the very small number of samples (89, 3.7%) with levels above this threshold; the parameters were unable to be well-estimated.

This study has several limitations. The first is that, due to the data collection process, we lack individual-level information on the mothers of the sampled newborns. This means that we cannot control for, or assess the contributing effects of, mothers’ age, race/ethnicity or other risk factors, such as the age of the house in which they live or the country in which they were born. Additionally, the samples collected over the 5 month period represent only 488 of the 935 ZCTAs in Iowa; a longer reporting period would have captured samples from a wider geographic distribution within the state.

This purely ecological analysis is also subject to flaws in the area-level data used as covariates. The American Community Survey replaced the long-form Census as the primary method by which data are collected on the US population. It contains many more items than the long-form, but it is a statistical sample with high margins of error, which increase when the spatial scale of analysis becomes smaller [84]. Despite this, however, ACS data are commonly used in public health and other analyses at scales smaller than counties because no similar datasets are available. As data were gathered in 2006, both population-level characteristics in Iowa as well as blood lead levels in newborns may have changed since then. Finally, this study did not examine variation in environmental lead sources, such as in soils or emitted from industrial or other sites, which have shown spatial relationships with birth outcomes [57].

Many studies based on grouped data tend to provide inaccurate individual-level inferences. In other words, ecological fallacy occurs when analyses based on grouped data lead to conclusions different from those based on individual data [85, 86]. In general though, the associations of the selected covariates with elevated blood lead levels in newborns were largely consistent with individual level data from other studies. Although the covariate percent foreign-born women in a ZCTA, which was dichotomized at the 75^th^ percentile (2.45% foreign-born), was not a significant predictor, inference was sensitive to the cut-point used for dichotomizing this severely right skewed covariate. For instance, cut-points at the 25^th^ percentile (0% foreign-born) and the 50^th^ percentile (0.7% foreign-born) led to inference suggesting that areas with higher proportions of foreign-born women were associated with decreased blood lead levels. Iowa is a largely rural state with a relatively homogeneous population (predominantly white); it has substantially fewer foreign born women than other parts of the US. Dichotomizing this variable, into a single foreign born woman of childbearing age present in a ZCTA or not, is possibly the reason behind the unanticipated finding of lower blood lead levels among births to foreign women. Having individual-level information on the mother, as previously mentioned, would be particularly beneficial in the case of this covariate.

While there are limitations in this study, it is the first to examine spatial patterns and ecological correlates of newborn blood lead levels in Iowa and one of only a few papers to explore blood lead levels in rural children. This study makes use of routinely collected dried blood spots to estimate the lead levels of blood in a randomly chosen population of Iowa newborns, finding that in a large share of the sample the levels of blood lead are above what is recommended by the CDC. Further, the distribution of blood lead levels and of elevated blood lead is similar across the rural and urban areas of Iowa. We also find that the characteristics of the population and types of housing where a mother resided at the time of the birth of her child are predictive of elevated blood lead concentrations, regardless of rural or urban residence. This suggests similar landscapes of risk and the need for physicians serving rural patients to be as aware of the dangers of lead poisoning as providers serving urban populations.

## Conclusions

The demographic characteristics identified in our analysis can guide the recommendation for testing of mothers in the prenatal period and infants at well-baby appointments in certain regions of the state where risk factors (old housing and low education) are present, potentially leading to prevention and earlier initiation of case management. Rural newborns exhibit similar levels of elevated blood lead as urban newborns, suggesting that exclusively focusing on risk in urban populations neglects a significant source of lead poisoning in US children.
