# Correction: High prevalence of elevated blood lead levels in both rural and urban Iowa newborns: Spatial patterns and area-level covariates

**DOI:** 10.1371/journal.pone.0196002

**Published:** 2018-04-12

**Authors:** Margaret Carrel, David Zahrieh, Sean G. Young, Jacob Oleson, Kelli K. Ryckman, Brian Wels, Donald L. Simmons, Audrey Saftlas

## Notice of Republication

This article was republished on January 30, 2018, as a Supporting Information file was published in error. Please download this article again to view the correct version.
